# Meetings reports

**DOI:** 10.1155/S1463924699000103

**Published:** 1999


'The fee reductions have been made possible by the continuous and successful efforts of management and staff to raise the quality of service and reduce unit costs since the Patent Office relocated to Newport, South Wales, in 1991'.

Details of the fee reductions are available on the Patent Office Web site at www.patent.gov.uk/snews/notices/red-fee.html. Copies of the statutory instruments are available from the Stationery Office Bookshops and from the Patent Office. They are *The Trade Marks (Fees) Rules 1998* (SI 1998 No 1776), £1.10; *The Registered Designs (Fees) Rules 1998* (SI 1998 No 1777), £1.10 and *The Patents (Fees) Rules 1998* (SI 1998 No 1778), £1.95).

*For information, contact Brian Caswell, The Patent Office, +44 (0)1633 814729 or Michael Binns, Prowse & Co, +44 (0)1372 363386.*

### 21st century requirements to monitor our environment prompts action by The Royal Society of Chemistry

Modern instrumentation has allowed us to push back the frontiers of detection such that we are able to determine incredibly small amounts of natural and anthropogenic pollutants and contaminants in our environment, whether they are in our homes, workplaces, cities, the countryside or the oceans. The fact that we can detect these pollutants in minuscule amounts does not necessarily mean that the levels present in the environment are harmful to our health or well being, but it does drive world-wide legislation on these substances. Therefore, there is a requirement to monitor, ascertain the sources, prevent the release, develop better detection methods and make properly assessed scientific judgements on the toxicity, exposure and risk assessment of the pollutants to which we are exposed in our daily lives.

The Royal Society of Chemistry has recognized the importance of these 21st century requirements, and that it is essential to promote and disseminate the knowledge of newly developed technologies for monitoring our various environments. Therefore, it is launching the *Journal of Environmental Monitoring (JEM)* which is dedicated to assessing exposure and health risks through the latest developments in measurement science. The journal, with the first issue due to be published in February 1999 and then bi-monthly thereafter, is unique in that it aims to publish all the relevant information on this subject area in one source.

This journal is intended for environmental and health professionals in industry, and officials from governmental and regulatory agencies as well as research scientists interested in the environment.

"I think the journal will be of interest to all analytical scientists involved with environmental monitoring issues. Currently at NIOSH, environmental monitoring is one of the key components of the National Occupational Research Agenda (NORA)". Dr E. R. Kennedy, NIOSH, Cincinnati, USA.

"Environmental contaminants are becoming the No. 1 public concern". Mr J. V. Dutton, Consultant, UK.

"The launching of a journal dedicated to environmental monitoring with some emphasis on legislative issues is an excellent idea". Dr. Philippe Quevauviller, European Commission, DGXII SM&T Programme, Belgium.

The Royal Society of Chemistry (RSC) is a learned society with a worldwide membership of 46 000. it has as its main objectives the advancement of the science of chemistry and its applications, and the maintenance of high standards of competence and integrity among practising chemists. The RSC markets a comprehensive range of high quality information products and services.

For further details contact: Harpal Minhas, The Royal Society of Chemistry, Thomas Graham House, Science Park, Milton Road, Cambridge CN4 4WF, UK. Tel: +44 (0)1223 432292; Fax: +44 (0)1223 420247; e-mail: jem@rsc.org; URL: www.rsc.org/jem

### Meetings reports

### Micromass host international seminar on the enabling role of MS

Micromass, the mass spectrometry people, hosted *MS in Proteomics & Drug Discovery: An International Seminar on the Enabling Role of MS*. The seminar, which was open to the public and incorporated Micromass' 1998 organic MS users' meeting, the first event of its kind to be organized by the company since its re-formation in 1996.

The seminar saw a move from central Manchester, the traditional home of the Micromass user meeting, to Shrigley Hall in Cheshire. From 18th to 20th October 1998, this impressive 19th century country mansion housed an international panel of invited speakers and Micromass users, presenting an insight into emerging MS-based strategies in proteomics and contemporary drug discovery/development. Speakers included Roland Annan (SmithKline Beecham, PA, USA), Walter Blackstock (Glaxo Wellcome, UK), Daryl Pappin (Imperial Cancer Research Foundation, UK) and Jeffrey Kiplinger (Pfizer, Groton, CT, USA).

Originally planned as a 'stand-alone' meeting to be hosted, earlier in 1998, by Glaxo-Wellcome, the proteomics sessions were, through unexpectedly popular demand, rescheduled at this larger venue. Protein identification using MS and bio-informatics is becoming routine in the quest for new targets, with known proteins being rapidly identified via comparison of MS data with genome, EST or protein databases. Unknown proteins require the use of partial sequencing by MS-MS for the construction of oligonucleotide probes or primers. Additionally, *de novo* sequencing of peptide fragments is being automated. Initially focusing on current achievements in protein characterization using Q-Tof™ MS-MS systems, the intention of this seminar was to present an assessment of what was currently achievable, the limitations and what problems remained to be solved.

Moving onto the pharmaceutical industry, the seminar investigated one of the most important changes taking place within it, i.e. the rapid expansion of discovery operations. MS contributes to this process in several ways, including the provision of structural support for synthetic chemistry, autopurification prior to screening and LC-MS in lead optimization. LC-MS(MS) is now being used to evaluate drug candidate metabolism and pharmacokinetics at both the early and late development stages. Discovery teams are increasingly being requested to focus on the survival of candidates to and beyond first testing in human subjects, touching off a strong push to move testing traditionally done in a development setting into the high throughput early discovery environment.

LC-TOF MS mass analysers (LCT/Q-Tof) were highlighted in view of their significant potential to leverage throughput and data quality in these challenging applications.

*For further information, contact Dawn Eaton, Micromass UK Limited, 3 Tudor Road, Altrincham, Cheshire WA14 5RZ, UK. Tel: +44 (0)161 282 9666. Fax: +44 (0)161 282 4400. E-mail: dawn.eaton@micromass.co.uk*

### ISLAR '98

Cooler temperatures and changing leaves not only signal the annual arrival of autumn, but also the return of the International Symposium on Laboratory Automation and Robotics (ISLAR). On 18–21 October 1998, the world's leading scientists and managers convened in Boston, MA, for an innovative experience at the 16th annual ISLAR symposium.

With over 130 podium and poster presentations, ISLAR '98 is the world's largest forum to learn about the latest developments in automation and robotics. Its unique format features both management and technical presentations, making it the most important industry-related conference of the year. World-renowned scientists and managers in the biotechnology, pharmaceutical, chemical and consumer products industries shared their insight on the importance of using the latest technology and strategies to increase productivity and reduce time to market.

The three-day program included a series of discussion groups, workshops, short courses and presentations developed for managers to exchange ideas and experiences. A new feature at ISLAR '98 was an Ultra High Throughput Screening session where scientists and managers from around the world discussed industry challenges, the new era of HTS, and how automation was being used as a critical component in the advancement of high-throughput screening.

Attendees and presenters at ISLAR '97 participated in a survey which resulted in several new sessions debuting at ISLAR '98, including the following.

- Assay miniaturization technologies
- The role of bioinformatics in pharmaceutical research
- The impact of automation on secondary screening
- Managing laboratory automation in pharmaceutical analysis
- Methods transfer and validation
- Inhalation applications

To help members of the media maximize their experiences at the symposium, ISLAR '98 featured a series of editorial roundtables focusing on drug discovery, analytical research and development, and quality control/quality assurance. The roundtable panels featured keynote speakers and symposium presenters to help facilitate discussions.

*For more detailed information and abstracts, access the ISLAR web site at http://www.islar.com. The next issue of* Journal of Automated Methods & Management in Chemistry *will feature abstracts from ISLAR '98*

